# Hæmorrhoids*Read before the Tri-State Medical Society of Alabama, Georgia and Tennessee.

**Published:** 1896-02

**Authors:** J. P. Stewart

**Affiliations:** Attalla, Ala.


### HÆMORRHOIDS.*

BY J. P STEWART, M. D., ATTALLA, ALA.

This is probably the most common of all surgical affections, the causes are so numerous and varying. They have been attributed to constipation and diarrhœa, to sedentary life and dysentery, to long rides and to long walks, to too much standing and to too much sitting, to overeating and to overstarving, to great exertion and to great rest, to straining, lifting, crying, coughing, sneezing, pregnancy, falling of the womb, gravel, smoking, drinking, etc., etc., too numerous to mention. But let the causes be what they may, we have them in all conditions and ages of both sexes.

They are of two varieties—external and internal—or those outside the sphincter and those inside. They originate in a varicose condition of the hæmorrhoidal plexus of vessels, the veins being the ones most usually affected.

The reason of this is simply that the hæmorrhoidal veins are large and valveless and ramifying the submucous substance of the lower gut, they are very liable to overdistention and congestion whenever the abdominal and pelvic circulation, from any cause, becomes sluggish. The hæmorrhoidal veins empty into the inferior mesentery, which is a radicle of the portal. So if we have, from any cause, a torpid liver, we have a weakened portal circulation, and a consequently sluggish hæmorrhoidal current and a definite cause for piles.

So much for cause. There is nothing more painful, disagreeable and uncomfortable than an attack of the piles. Let a small hæmorrhoid form just within the sphincter, or involving the sphincter so far as to feel like there was something just inside that wanted at all times to come out, and the remembrance of the last attempt at defecation when it did pop out, the pain, the strain, the weak, sick sensation, the heroic efforts by which with aid of the bottle of goose greese and perseverance it was finally put back, the throbbing, burning sensation that followed for hours, and the continued uneasy feeling, all comes back with the desire to defecate again. Oh, the horrors of the piles! No wonder we meet so many men carrying buckeyes in their

*Read before the Tri-State Medical Society of Alabama, Georgia and Tennessee.

pockets, who laugh at superstition and smile scornfully at faith cures.

The suffering is intense. It has killed many. It has run many distracted. It has caused abortions and miscarriages. It has made many lose their jobs and many more their religion. It unfits a person for business, society or medicine. In fact, piles are a holy terror.

The diagnosis is easy. A little stretching of the sphincter ani and there you are. It's piles or fissure, or ulceration, at a glance.

The treatment is not quite as easy. It must be such as has an eye to the defecation that is sure to follow before the healing is done. There are several methods of operation—injections, ligation, and clamp, and cautery. I will not go into detail of all these, but give you my treatment.

A patient comes to me with piles. I examine him; that is, I place him or her in Sims' position on my operating table, give him or her chloroform, if necessary, and open up the anus. If I find one or two little hæmorrhoids, I do not operate at all, but treat them in the following manner: I give them a rectal injection of red gum, fluid extract, and glycerine, to be used after each action, and a suppository of coca butter and iodoform once or twice a day. I always stretch the sphincter while I am making the initial examination. I give a laxative if the bowels have been costive; if the patient is a sufferer from diarrhœa and dysentery, I give opium and ipecac. Should I find the tumors large, numerous and tender, or small and quite sensitive, I dissect away the mucous membrane from over them, clamp them and use the actual cautery, following it with the same treatment as before mentioned.

I have yet to record a single case in which I have failed to make a cure, as far as I now know.

